# Development of a culturally targeted chatbot to inform living kidney donor candidates of African ancestry about *APOL1* genetic testing: a mixed methods study

**DOI:** 10.1007/s12687-024-00698-8

**Published:** 2024-02-13

**Authors:** Elisa J. Gordon, Jessica Gacki-Smith, Matthew J. Gooden, Preeya Waite, Rochell Yacat, Zenab R. Abubakari, Debra Duquette, Akansha Agrawal, John Friedewald, Sarah K. Savage, Matthew Cooper, Alexander Gilbert, Lutfiyya N. Muhammad, Catherine Wicklund

**Affiliations:** 1 Department of Surgery, Center for Biomedical Ethics and Society, Vanderbilt University Medical Center, 1161 21St Avenue South, D-4314 Medical Center North Nashville, Nashville, TN 37232-2730 USA; 2 Center for Health Services and Outcomes Research, Northwestern University Feinberg School of Medicine, Chicago, IL USA; 3 Medstar Georgetown Transplant Institute, Georgetown University Hospital, Washington, DC USA; 4 Medicine, Cardiology Division, Northwestern University Feinberg School of Medicine, Chicago, IL USA; 5 Division of Nephrology and Hypertension, Northwestern University Feinberg School of Medicine, Chicago, IL USA; 6 Invitae Corporation, San Francisco, CA USA; 7 Froedtert Hospital Center for Advanced Care, Froedtert Memorial Lutheran Hospital Children’s Hospital of Wisconsin Medical College of Wisconsin, Milwaukee, WI USA; 8 Children’s Hospital of Wisconsin, Milwaukee, WI USA; 9 Medical College of Wisconsin, Milwaukee, WI USA; 10 Froedtert Hospital Center for Advanced Care, Milwaukee, WI USA; 11 Department of Preventive Medicine, Division of Biostatistics, Northwestern University Feinberg School of Medicine, Chicago, IL USA; 12 Department of Obstetrics and Gynecology, Northwestern University Feinberg School of Medicine, Chicago, IL USA

**Keywords:** Digital health technologies, Equity, Adaptation, Black, African American, Genetic counseling, Community engagement, Evaluation, Nephrology, Transplantation, Focus groups, Informed consent, Patient-centered, Scaling

## Abstract

Clinical chatbots are increasingly used to help integrate genetic testing into clinical contexts, but no chatbot exists for *Apolipoprotein* L1 (*APOL1*) genetic testing of living kidney donor (LKD) candidates of African ancestry. Our study aimed to culturally adapt and assess perceptions of the Gia® chatbot to help integrate *APOL1* testing into LKD evaluation. Ten focus groups and post-focus group surveys were conducted with 54 LKDs, community members, and kidney transplant recipients of African ancestry. Data were analyzed through thematic analysis and descriptive statistics. Key themes about making Gia culturally targeted included ensuring: (1) transparency by providing Black LKDs’ testimonials, explaining patient privacy and confidentiality protections, and explaining how genetic testing can help LKD evaluation; (2) content is informative by educating Black LKDs about *APOL1* testing instead of aiming to convince them to undergo testing, presenting statistics, and describing how genetic discrimination is legally prevented; and (3) content avoids stigma about living donation in the Black community. Most agreed Gia was neutral and unbiased (82%), trustworthy (82%), and words, phrases, and expressions were familiar to the intended audience (85%). Our culturally adapted *APOL1* Gia chatbot was well regarded. Future research should assess how this chatbot could supplement provider discussion prior to genetic testing to scale *APOL1* counseling and testing for LKD candidate clinical evaluation.

**Supplementary Information:**

The online version contains supplementary material available at 10.1007/s12687-024-00698-8.

## Introduction

As personalized medicine gains greater momentum, the proliferation of genetic testing has enabled more patients to make value-based preventive treatment decisions. An essential component of genetic testing is the provision of counseling by genetic counselors before and after the test. However, the limited number of genetic counselors (National Society of Genetic Counselors 2022) and gap in physician knowledge of genetics to make referrals to genetic counselors (Rasouly et al. 2023) hinder patients’ access to genetic services. Increasing patients’ access to genetics expertise can be addressed by alternate delivery models that leverage innovative technologies to scale genetic information delivery, such as clinical chatbots (Schmidlen et al. 2019; Nazareth et al. 2021).

Clinical chatbots, a type of “conversational agent,” use a text-based interface to simulate conversation with the user via smartphones, or other digital devices. (Allouch et al. 2021) Chatbots are invaluable for streamlining the repetitive foundational information-giving responsibilities of providers, thereby providing more time for interpreting test results and addressing emotional dimensions of patients’ decision-making (National Society of Genetic Counselors 2022; Nazareth et al. 2021). Chatbots are particularly useful for delivering information about genetic conditions given patients’ limited health literacy in genetics, the heightened challenge of making preference-based treatment decisions in the context of uncertainty, and the extensiveness of genetic information delivered to patients (Bombard et al. 2022).

Chatbot use can be applied across the genomics pathway, including pre- and post-test counseling, and management (Bombard et al. 2022). Chatbots have been used for many purposes (e.g., to aid in decision-making about genetic testing (Siglen et al. 2022), provide secondary findings to patients undergoing genomic sequencing (Ireland et al. 2021), provide informed consent for genetic testing (Schmidlen et al. 2019), and enable self-diagnosis (Fan et al. 2021)). Chatbot use can improve knowledge of genetic testing, informed decision-making, physical activity, self-care, and health literacy (Ireland et al. 2021; Luca et al. 2023; Owens et al. 2019; Bickmore et al. 2013; Dworkin et al. 2019; Pereira and Diaz 2019).

Given their utility, healthcare institutions are increasingly using chatbots in genetic services delivery, like Gia® (Genetic Information Assistant), the Invitae® Health Insurance Portability and Accountability Act (HIPAA)-compliant chatbot (Schmidlen et al. 2019; Allouch et al. 2021; Ray 2018). However, integrating chatbots into the clinical context may be challenging to embed within the workflow and ensure patients understand the content and usability (Nazareth et al. 2021). Research shows that patients embrace chatbots (Schmidlen et al. 2018; Ponathil et al. 2020).

In the context of kidney transplantation, which is predominated by organ scarcity, evaluating patients’ eligibility is essential for providing safe and equitable treatment. Genetic testing comprises an important component of the evaluation process through screening, diagnosis, management, and prognostication of kidney disease. It also provides helpful information for risk stratification in potential living kidney donor (LKD) candidates (Caliskan et al. 2022). Given the risk of inherited kidney disease in LKDs post-donation, guidelines recommend that LKD candidates undergo genetic testing in certain clinical situations (Lentine et al. 2017).

Testing for *Apolipoprotein* L1 (*APOL1*) risk variants in LKD candidates is one such consideration currently under debate (Freedman and Poggio 2021; Kumar and Locke 2021). The presence of two risk variants is significantly associated with kidney failure in the general population, prominently among individuals of African ancestry (Parsa et al. 2013). Evidence from observational studies and case reports suggests that genetic factors contribute to an elevated risk of kidney failure post-donation among LKDs of African ancestry (Muzaale et al. 2014). *APOL1* risk variants are most prevalent among sub-Saharan African, West African, and Black/African American populations, while also found among Brazilian, Jamaican, Granadian populations (11–22%), and least prevalent in Native American, Peruvian, Belizean populations (1–5%), among other populations (Nadkarni et al. 2018). *APOL1* risk variants occur in Hispanic/Latino populations at < 1% (Kramer et al. 2017). “Black” and “African American” comprise terms of social identity and, as social constructions, such terms may not align precisely with one’s genetic ancestry, as research shows (Lazarin et al. 2013). However, approximately 36% of all individuals identifying as Black or African American in the USA carry *APOL1* risk variants, which is larger than any other ethnic or racial group in the USA (Friedman and Pollack 2011). We use “African ancestry” to refer to individuals with African descent, including Black and African American individuals who are born outside of or born inside the USA, respectively. We henceforth use “Black” inclusively to designate a shared cultural and historical global identity (Simms 2018).

The lack of evidence demonstrating a causal link between *APOL1* risk variants and LKDs’ graft outcomes and ethical issues of *APOL1* testing have given some transplant professionals pause. Given the available evidence, the importance the transplant community places on protecting living donor’s safety (Tan et al. 2015), and the 2017 KDIGO Clinical Practice Guideline on the Evaluation and Care of Living Kidney Donors which recommends “…considering *APOL1* genotyping in LD candidates with sub-Saharan African ancestors” have justified the implementation of some degree of *APOL1* testing into clinical practice for many surveyed transplant centers (Gordon et al. 2018; Doshi et al. 2021). Accordingly, it is essential that centers performing *APOL1* genetic testing do so in an ethically and culturally targeted manner to avoid reinforcing systemic racism (Smith et al. 2023).

The presence of two *APOL1* risk variants can comprise a contraindication to living donation, requiring careful counseling and consent of LKD candidates (Riella and Sheridan 2015). However, some transplant physicians lack the knowledge to educate LKDs about *APOL1* testing and results for donation, and few transplant programs leverage genetic counselors to aid in counseling (Rasouly et al. 2023; Gordon et al. 2018). The optimal implementation of genetic counseling and testing in LKD evaluation remains to be developed (Caliskan et al. 2022). To date, no chatbot has been used in the LKD context or adapted to *APOL1*.

Underserved populations have disproportionately less access to genetic services and digital health technologies, like chatbots. Relatively little is known about how Black patients perceive chatbots for genetic testing. Studies examining chatbot use in Black individuals demonstrated acceptability and efficacy (Owens et al. 2019; Bickmore et al. 2013; Dworkin et al. 2019). Increasing equity in accessibility requires designing chatbots that foster genetic literacy and trustworthiness (Bombard et al. 2022; Fan et al. 2021). To date, no studies have adapted chatbots to Black individuals who may share cultural worldview, to enhance receptivity to their use. Adapting evidence-based interventions to the self-ascribed Black population, an underrepresented population that comprises the majority of those who have *APOL1* risk variants and identify as having African ancestry, requires addressing culturally shared beliefs regarding distrust of healthcare providers and genetic testing (Gordon et al. 2018; Gordon et al. 2019). Culturally targeted care can increase Black patients’ knowledge about donation and LKDs rates (Arriola et al. 2014). In this study, we adapted Gia to the *APOL1* genetic testing context, and culturally adapted (Barrera et al. 2017). Gia to LKD candidates of African ancestry. Our intent was to scale genetic testing by making Gia available to LKD candidates of African ancestry to facilitate making informed decisions about *APOL1* testing and ultimately living donation.

## Materials and methods

### Study design

This sequential mixed methods study involved focus groups and post-focus group surveys about our *APOL1* Gia chatbot (Creswell and Plano Clark 2007). Data collection occurred in an iterative process, whereby changes based on focus group feedback were applied to Gia for review in subsequent focus groups to maximize user-centeredness. Focus groups and surveys involved LKDs, community members, and living donor kidney transplant recipients of African ancestry.

Our study was conducted as part of a larger study evaluating the integration of a culturally competent *APOL1* genetic testing program into LKD candidate evaluation at Northwestern University (NU) in Chicago, IL and Georgetown University (GU) in Washington, DC (Smith et al. 2023). The study was approved by the Institutional Review Boards at NU (STU00214038) and GU (STUDY00003752); NU served as the Institutional Review Board of record for GU. We used the Consolidated Criteria for Reporting Qualitative Research for quality reporting of qualitative studies (Tong et al. 2007).

### Chatbot development process

#### Research team

Our research team adapted Gia to present information on *APOL1* testing for LKDs of African ancestry. The research team included social scientists, genetic counselors, and transplant nephrologists from NU and GU, genetic counselors from Invitae, and a Scientific Advisory Board comprised of experts in nephrology, ethics and genetics, and race and genetics.

#### Community Advisory Board (CAB)

We established a nine-member, Chicago-based CAB, comprised of one living kidney donor, two kidney transplant recipients, and community leaders of African ancestry from organizations with expertise in kidney disease, organ donation and transplantation, and Black cultural values and community needs. During seven meetings between June 2021 and January 2022, CAB members provided feedback on Gia’s content, appearance, wording, tone, and cultural sensitivity.

#### Adaptation

Gia allows users to interact by selecting one of the programmed response options to progress through the chat. Gia has one main chat flow path. Users can select additional information in optional chat flow paths and return to the main path, based on their selected responses. Thus, the information presented in Gia is based on the user’s learning needs.

The research team adapted Gia in two ways: (1) topically, on *APOL1* and living donation, and (2) culturally, to the intended group it aimed to serve—Black LKD candidates. Overall, adaptation entailed presenting *APOL1* information in a conversational, neutral, unbiased, and culturally targeted manner. The topics, data, and resources included were obtained from peer-reviewed articles, the National Kidney Foundation, the National Institute of Diabetes and Digestive and Kidney Diseases, our research team, and CAB (Parsa et al. 2013; Gordon et al. 2018). Gia included information on basic genetic concepts and kidney disease, how *APOL1* relates to kidney disease, prevalence of *APOL1* risk variants, what *APOL1* test results mean in relation to kidney disease and donation, reasons for *APOL1* testing, the logistics of undergoing *APOL1* testing, and the Genetic Information Nondiscrimination Act.

Cultural adaptation also involved making health communication culturally targeted, which refers to “a single intervention approach for a defined population subgroup that takes into account characteristics shared by the subgroup’s members,” in order to increase receptivity and acceptance of messages (Kreuter and Skinner 2000). This step is important because ensuring that health educational programs are relevant to the intended population is a core principle of public health practice (Glanz et al. 1997).

We used several strategies including (1) stakeholder engagement (i.e., involving members of the intended group, and the scientific advisory board, to provide input on chatbot development and insights into cultural dimensions); (2) addressing “deep structure” (i.e., embedding *APOL1* content, design, and appearance of Gia in the context of shared cultural values, norms, and beliefs of individuals of African ancestry) to generate meaning about and enhance the salience of health messages; (3) presenting epidemiological data on the health impact of *APOL1* variants on individuals of African ancestry (i.e., “13 out of every 100 (13%) people who identify as Black or African American have 2 *APOL1* risk variants”) to raise awareness and perceived relevance of *APOL1* and promote taking preventative action; (4) addressing “surface structure” by using an avatar representative of the intended group to make the chatbot seem more familiar and comfortable; and (5) ensuring that the chatbot used language and phrasing commonly used by the intended group (Resnicow et al. 1999).

#### Scope

We adapted Gia as part of a multi-level intervention to integrate *APOL1* testing and counseling into LKD candidate evaluation. Gia was designed for use by LKD candidates as a supplemental tool providing foundational information prior to their clinical evaluation with the transplant nephrologist.

### Data collection

#### Focus groups

Eligible individuals included English-speaking adults (age 18 + years) of self-identified African ancestry who were LKDs, community members, or living donor kidney transplant recipients. Participants were recruited via two methods: (1) LKDs or transplant recipients who donated or received a kidney within the previous 15 years were identified by medical record review, mailed a letter and/or email, and called a week later to screen for eligibility; and (2) CAB members distributed study flyers; interested community members contacted the research staff. All participants provided verbal informed consent.

Focus groups were conducted remotely and led by female moderators [PW and RY] trained in focus group facilitation by a seasoned qualitative researcher [EJG]. Moderators used standard procedures to direct the discussion (Krueger 1994), published elsewhere (Smith et al. 2023). Focus groups began by providing background information about living kidney donation, relationship between *APOL1* and kidney disease among individuals of African ancestry*,* and the purpose of Gia to ensure that participants had sufficient knowledge to respond to questions. Focus group questions evolved based on iterative updates to Gia. Thereafter, we provided participants the link to Gia to use for 8 min, and then invited initial impressions about their experience. We sought feedback on Gia’s cultural sensitivity, content, design, function, organization, appearance, and tone. Research staff took field notes. After each focus group, participants completed an online survey on selected, relevant attitudes about Gia’s cultural sensitivity (Guidry and Walker 1999) and demographic questions. Each focus group lasted approximately 78 min [range: 37–140] and was audio-recorded. Participants were compensated with a $50 e-gift card for their time.

### Data analysis

#### Thematic analysis

Focus group audio recordings were transcribed verbatim and analyzed for emergent themes using the constant comparison and inductive and deductive coding methods (Guest et al. 2012). An a priori list of defined codes corresponding to focus group questions was developed. All responses were coded using NVivo qualitative software (QSR International). The research team [EJG, MJG, PW, JG-S, RY, ZA] used a rapid, iterative process to independently review and openly code the first set of transcripts (Keith et al. 2017). Thereafter, the research team revised the coding scheme by adjusting for new responses, with modified codes applied to prior transcripts, until reaching thematic saturation (when no new themes emerged). Transcripts were coded after establishing inter-rater reliability (kappa > 0.80) (Guest et al. 2012). We reviewed all segments pertaining to each code to develop analytic summaries that synthesized emergent patterns (Keith et al. 2017).

#### Descriptive statistics

Post-focus group survey and demographic data were analyzed using descriptive statistics. Medians, interquartile range (IQR), and frequencies were calculated using IBM SPSS Statistics for Windows, version 28. Fisher’s exact test was used for categorical variables, and Wilcoxon rank sum test was used for continuous variables.

## Results

### Demographics

Ten focus groups (Chicago, IL *n* = 5; Washington, DC *n* = 5) were conducted with *n* = 54 participants. Focus group participant composition included LKDs (*n* = 5 groups, *n* = 25 participants, 46%), community members (*n* = 4 groups, *n* = 23 participants, 43%), and living donor kidney transplant recipients (*n* = 1 group, *n* = 6 participants, 11%). Most participants were female (72%), and the mean age was 44 years (Table 1, Supplemental Table 1). Characteristics differed significantly by site, with NU including more younger, male, and publicly insured participants than GU.

**Table 1:** Focus group participant demographic characteristics

Characteristic	Total *N* = 54 *n* (%)	NU *n* = 30 *n* (%)	GU *n* = 24 *n* (%)	*p*-value
Age, years, median [IQR]	47.00 [28.50, 55.75]	35.0 [26.00, 52.25]	50.5 [44.25, 56.25]	0.020
Sex				0.034
Female	39 (72.2)	18 (60.0)	21 (87.5)	
Male	15 (27.8)	12 (40.0)	3 (12.5)	
Highest education level				0.125
High school graduate or Some college	16 (29.6)	6 (20.0)	10 (41.7)	
College graduate	28 (51.9)	19 (63.3)	9 (37.5)	
Post graduate degree (MA, PhD, MD, DO, etc.)	10 (18.5)	5 (16.7)	5 (20.8)	
Marital status				0.283
Married/Domestic partner/Civil union/ Living with partner	29 (53.7)	14 (46.7)	15 (62.5)	
Separated or divorced/Never married/Single/Widowed	25 (46.3)	16 (53.3)	9 (37.5)	
Ethnic identity				0.245
Hispanic/Latino	3 (5.6)	3 (10.0)	0 (0)	
Not Hispanic/Latino	51 (94.4)	27 (90.0)	24 (100)	
Race				n/a
African American/Black	52 (96.3)	29 (96.7)	23 (95.8)	
Multi-racial	2 (3.7)	1 (3.30)	1 (4.2)	
Employment status				0.019
Employed full-time	33 (61.1)	14 (46.7)	19 (79.2)	
Employed part-time	14 (25.9)	12 (40.0)	2 (8.3)	
Not employed/Homemaker/Disabled	7 (13.0)	4 (13.3)	3 (12.5)	
Annual income				0.013
Less than $34,999	7 (13.0)	6 (20.0)	1 (4.2)	
Between $35,000 and $74,999	21 (38.9)	14 (46.7)	7 (29.2)	
Between $75,000 and $94,999	7 (13)	5 (16.7)	2 (8.3)	
More than $95,000	19 (35.2)	5 (16.7)	14 (58.3)	
Primary health insurance coverage				0.002
Private health insurance	42 (77.8)	19 (63.5)	23 (95.8)	
Medicaid/Medicare	10 (18.5)	10 (33.3)	0 (0)	
None	2 (3.7)	1 (3.3)	1 (4.2)	
Health literacy				0.620
Adequate	50 (92.6)	27 (90.0)	23 (95.8)	
Inadequate	4 (7.4)	3 (10.0)	1 (4.2)	
Own a working smartphone				
Yes	54 (100.0)	30 (100.0)	24 (100.0)	
Send or receive text messages				
Yes	54 (100.0)	30 (100.0)	24 (100.0)	
Time spent using Internet per week				1.000
Less than or equal to 20 h	27 (50.0)	15 (50.0)	12 (50.0)	
More than 20 h	27 (50.0)	15 (50.0)	12 (50.0)	
Registered as an organ donor				0.772
Yes	36 (66.7)	19 (63.3)	17 (70.8)	
No	18 (33.3)	11 (36.7)	7 (29.2)	

### Culturally adapting Gia

Three interrelated themes emerged about making Gia culturally targeted to Black community perspectives: (a) ensuring transparency; (b) designing Gia as an informative, rather than persuasive, tool; and (c) avoiding stigma related to *APOL1* and kidney donation within the Black community. Representative, illustrative quotes are presented in Table 2.

**Table 2:** Representative illustrative quotations about Gia-*APOL1* and using chatbots by major theme and subtheme

Themes/	Illustrative quotations
Reactions to Gia	
	“Not to sound like a broken record, but I agree with everyone. I think the presentation was informative. It was easy to read, it flowed, it provided information and I think it is a[n] excellent tool to present to someone who is considering donating. (GU FG#6, Female)
	“The most important part for me was knowing that the genetic testing results are going to be kept safe.” (NU FG#3, Female)
Culturally targeted	
Transparency	“… [Gia has] information that is accurate and is, so you just want the information that’s given you to be accurate and to be truthful…” (NU FG#2, Female)
	**“**I would suggest a statistic of how many African Americans have donated, but I don’t know if that’s a good number to put out there, just to make others comfortable, and let them know that there are African Americans that donate.” (NU FG#4, Female)
	“That's the main option. I think if you're expressing how many people who've actually done it, and are living, and surviving, and things like that because most people jump into it because it's a relative. They don't necessarily do it because, ‘Oh, that's what I really want to do.’ But they do it because they love their family members, and they want them to live. So I think open transparency is a good way of marketing it.” (GU FG#1)
	“That's a tough one because the reality of it is that, there, like you said, there's a level of distrust. And I mean, the only way that you can really get away from that is, I agree with the stories and other testimonials, but I think what's important is that has to be a clear message being sent out that, transparency is there.” (GU FG#10, Male)
Informative	“All of this, just more information, needs to be at the beginning, and that may even be because if someone just knows it’s a saliva test versus a blood test, you know, ‘OK, I might be willing to do that.’ Then you say, well, then it’s no cost to you, if you’re willing to do this as a donor. That might nudge them on, you know, ‘Well, let me just see what’s going on.’ And not only for them being a donor. [Gia] might be as an information tool for their health as well.” (NU FG#3, Female)
	“Yes, I think Gia should try to convince our, the consumers of the information, that—who are the people with African ancestry—that it is OK to have a gene variant because as a result of adapting to the African savannah, people—or rather, their ancestors—tried to change their genetics [unintelligible] to that environment, which is not a bad thing. So I think Gia should try to convince them that even though this problem—or rather, the risk variant to the APOL1—is basically for the people with the African ancestry, it is not a bad thing. It is not a bad thing to them. Try to convince them, try to not make them seem inferior to the White [LKDs]. Yeah, that’s my take.” (NU FG#2, Male)
Not persuasive	“Yeah. I agree. I think this should be informative, but it shouldn't be like it's a campaign, or you're trying to push people. Give the information. Sometimes a lot is too much. And as I think somebody said before you, you don't want to feel like you're trying too hard, and then you're turning people off. I think the way it is, I don't feel like there's any stereotypes or anything in the presentation, but I don't think you need to add any more as a way of persuading.” (GU FG#6, Female)
	“What I was saying is I think that it's better to keep it the way it is. So it don't seem like you're trying to beg one race or the other to do it. So I think what you're doing is being informative. Rather be informative and let it be that because you don't want to feel like it's swaying one way, or I really have to get this group in here. You don't want to do that.” (GU FG#6, Female)
Avoiding stigmatizing language	“I know that research came up with these numbers and there was much research and data and all of that, that came up with these percentages. But it's always been, not a sore spot, but it always gives me pause. When I see that, Black people are more likely to have this, this, this, or that. And, White people are less likely to have this, this, or that. It still gives a negative connotation. Those comparison numbers, percentages, it still gives a negative connotation to who I am as a Black person.” (GU FG#8, Female)
	“I think it's gotten better, but some of the older generation, there is stigmas because of things that have happened at other hospitals, where things have happened and there are instances where it's true and they hold onto that. So if they're in the younger generation's ear, they've kind of planted that seed and it perpetuates from [generation] to generation. So I think having people who have already done [living donation] and gone through, having them available for people to talk to maybe, would be helpful.” (GU FG#10, Female)
	“Do you think it would be helpful to have information in Gia about the benefits of kidney donation? Because even though I'm a donor, I still feel that there is a very big stigma within the community about organ donation.” (GU FG#5)

#### A. Transparency

Participants explained that transparency meant presenting information in Gia accurately and comprehensively (including positive and negative information), and reflecting LKDs’ experiences. Transparency was considered beneficial for ensuring that LKD candidates of African ancestry feel comfortable proceeding with the LKD evaluation process, and for dispelling myths in the Black community about genetic testing and donation. Participants recommended the addition of testimonials or “stories” of Black LKDs discussing their *APOL1* test results and their health several years post-donation. LKD testimonials could help to normalize the idea of Black individuals donating and enhance LKD candidates’ comfort with *APOL1* testing and the prospect of donating.

We addressed the need for transparency in several ways. First, we fostered trust by including questions and answers in Gia that were community-driven. Originally, the question “How long has the medical community known about *APOL1*?”, which derived from our prior research (Gordon et al. 2018), was set up as an optional pathway. However, our CAB recommended making this a mandatory part of the chat that every LKD candidate would see, so that Gia did not come across as hiding information from the user.

Second, given the well-documented importance placed by people of African ancestry on protecting patient privacy and confidentiality, we addressed the disposition of *APOL1* test results by stating in Gia that “Your test results will be shared with you, your transplant team, the genetic testing lab, and with authorized research personnel only.” During an early focus group, a participant suggested greater transparency about who “authorized research personnel” are. Accordingly, we added a hover-over definition to this term: “This is the person who asked for your informed consent for this study, the principal investigator of the study, and other staff working to collect and analyze study data.”

Third, Gia highlighted the usefulness of genetic testing in LKD candidate evaluation to show people of African ancestry that this genetic test will help their donation process. Specifically, Gia states: “Genetic testing for *APOL1*, along with all the other donor tests, helps the transplant team better understand your personal risk of getting kidney disease after donating. Genetic testing for *APOL1* may also help you feel better prepared and better informed about deciding about donating.” The usefulness of genetic testing in LKD evaluation is highly relevant for Black LKD candidates because Gia presents that information to the donor to make them aware of how genetic testing fits into the entire donor evaluation process.

#### B. Informative

Participants emphasized the importance of making Gia “informative” to educate Black LKD candidates, rather than aim to convince them to pursue genetic testing.

We addressed the need for Gia to be informative in several ways. First, we included a pathway option “Why do *APOL1* risk variants occur mostly in people of African ancestry?”. To answer this question, Gia stated: “The *APOL1* risk variants evolved about four thousand years ago in Sub-Saharan Africa to protect against sleeping sickness.” That statement was well received by focus group members because it recast *APOL1* risk variants in a positive light by highlighting a strength of people of African ancestry. We piloted the additional explanation of why *APOL1* gene variants existed primarily in people of African ancestry by saying how ancestors to modern Europeans had migrated out of Africa, prior to when *APOL1* evolved. However, CAB members and focus group participants suggested that we remove this additional information. CAB members expressed that the information may not be received well or may come across as insensitive because, for example, White Europeans had the choice to leave Africa. One focus group member remarked, “I don’t think [the additional information] is necessary to have. Because [the additional information] does make [the chatbot] a little longer and I don’t think that donors necessarily need that information.”

Second, Gia describes the *APOL1* testing process: “*APOL1* testing is voluntary, free to study participants, and involves taking a saliva sample.” These three points were initially presented separately and appeared later in the chat. When participants came upon the information, they consistently indicated that the information answered questions early in the chat. As a result, this statement was deliberately presented early in Gia. Presenting this information early fostered trust by addressing user-driven information needs.

Third, Gia describes how discrimination is prevented: “There is a law called GINA (Genetic Information Nondiscrimination Act) that protects patients against discrimination by health insurance providers and employers based on genetic information.”

#### C. Avoiding stigma

A pervasive concern was the need to reframe *APOL1* in a positive manner, and to minimize comparisons between Black and White LKDs, which were perceived as off-putting. Avoiding stigma entailed framing the higher frequency of *APOL1* risk variants in the population of those with African ancestry in a positive way so that LKD candidates did not feel “inferior to the White [LKDs].” Participants spoke about addressing the stigma related to living donation within the Black community by presenting statistics about kidney donation and the benefits in Gia. Participants suggested adding testimonials and statistics on “those who have donated… as well as those who are really on a waiting list, waiting” who are Black as it would be “pretty impactful” information that could encourage people in the Black community to become living donors.

### Perceptions of Gia

Participants’ ratings of Gia’s cultural targeting indicated that our adaptations were appropriate (Table 3). Most participants “agreed” or “strongly agreed” that Gia is “neutral and unbiased” (82%), trustworthy (82%), and that “words, phrases, and expressions are familiar to the intended audience” (85%).

**Table 3:** Focus group participants’ attitudes about cultural sensitivity of the *APOL1* Gia chatbot, *N* = 54

	Strongly agree	Agree	Neutral	Disagree	Strongly disagree
	*n* (%)	*n* (%)	*n* (%)	*n* (%)	*n* (%)
GIA is neutral and unbiased. That is, GIA is neither in favor of nor against getting *APOL1* testing	29 (53.7)	15 (27.8)	8 (14.8)	1 (1.9)	1 (1.9)
GIA is trustworthy	26 (48.2)	18 (33.3)	9 (16.7)	1 (1.9)	0 (0)
Information is presented in a way that is easy to understand and follow	33 (61.1)	15 (27.8)	1 (1.9)	5 (9.3)	0 (0)
The medical terms are understandable to the intended audience	30 (55.6)	21 (38.9)	2 (3.7)	1 (1.9)	0 (0)
The words, phrases, and expressions are familiar to the intended audience	24 (44.4)	22 (40.7)	6 (11.1)	2 (3.7)	0 (0)
The words, phrases, and expressions are free from stereotypical meaning	28 (51.9)	23 (42.6)	2 (3.7)	1 (1.9)	0 (0)
The messages are linked to sources credible to the intended audience	23 (42.6)	20 (37)	9 (16.7)	2 (3.7)	0 (0)
The educational materials are culturally sensitive to ethnic minority communities	12 (22.2)	22 (40.7)	14 (25.9)	5 (9.3)	1 (1.9)
The graphics enhance the learning process	16 (29.6)	20 (37)	11 (20.4)	7 (13)	0 (0)

We asked focus groups about their initial impressions immediately after using Gia and at the end of the focus group. Participants consistently regarded Gia positively and described Gia as follows: “informative,” “engaging,” “interactive,” “straightforward,” “personable,” “user-friendly,” and “excellent.”

### Incorporating CAB and focus group feedback

The CAB and focus groups provided extensive feedback and recommendations for improving Gia chatbot content, design, functionality, and appearance. Supplemental Table 2 presents the prominent, commonly shared recommendations, whether each recommended change was implemented, the rationale for not implementing the change, if applicable, and how Gia was changed in response to the recommendation.

We incorporated as many suggestions as possible to be user-centered. For example, several participants said that the tone of some response options (i.e., “I see” or “Okay”) could be perceived as “abrupt” or “curt” within the Black community. Alternative responses (i.e., “That makes sense” and “I understand”) were considered appropriate in subsequent focus groups and used instead. Additionally, participants across several focus groups desired supplemental resources in Gia about Black LKD testimonials of donation experiences and self-care strategies, which led to the inclusion of links to websites and educational videos. To make Gia sensitive to LKD candidates, CAB members suggested including a strong phrase to convey gratitude to the LKD candidate for considering donation, which became: “Thank you very much for considering donating the gift of life.”

However, not all suggestions were accommodated due to technical constraints, study cost constraints, idiosyncratic or contradictory feedback, or our intended use of Gia in clinical practice. For example, many participants suggested that Gia provide an audio function for those who cannot or prefer not to read, but implementation of this feature was not in scope for this project. Some participants recommended including information about whether the LKD candidate would be able to donate with two *APOL1* risk variants. We deliberately did not include this information because Gia’s intent was to provide foundational information about *APOL1*, and to relegate the shared decision-making process to the transplant nephrologist’s meeting with the LKD candidate.

## Discussion

This is the first study to culturally adapt a chatbot to individuals of African ancestry and to the *APOL1* genetic testing context. We found that individuals of African ancestry were highly receptive to our chatbot for informing LKD candidates about *APOL1* genetic testing in a culturally targeted manner. By adapting Gia to the cultural considerations of individuals of African ancestry through community engagement, our chatbot overcomes limitations of prior studies which designed chatbots for the end user rather than with the end user (Kramer et al. 2020).

Three themes that emerged from making Gia culturally targeted to LKD candidates of African ancestry included making content transparent, informative, and non-stigmatizing. Transparency entailed providing balanced statistical information, while being informative entailed providing testimonials of Black LKDs, which fostered trustworthiness. A pervasive concern was the importance of reframing *APOL1* in a positive manner (i.e., as an evolutionary protection from sleeping sickness), and to minimize comparisons between Black and White LKDs. Our prior research among Black LKDs similarly found that framing *APOL1* in a positive manner was important to avoid stigmatizing language about Black individuals and their health (Gordon et al. 2019). Other research suggests that chatbots could become more trustworthy by disclosing the data sources, accuracy of predictions, and how diagnostic reports are produced (Fan et al. 2021). Our participants’ positive perceptions of Gia suggests that our chatbot was viewed as trustworthy and holds promise for use in the genetic testing and counseling process.

Bombard et al. suggest, digital genomic tools can reinforce disparities in access to genetic services if tools are not developed in a trustworthy manner (Bombard et al. 2022). Chatbots are appealing for increasing patients’ access to genetic information, thereby fostering equity in precision medicine (Nazareth et al. 2021). Ensuring that Gia’s content and design are culturally targeted for use among LKDs of African ancestry enhances Gia’s ability to reach and resonate with people who have been traditionally underserved, and are most at risk of kidney disease and *APOL1* risk variants, and thereby increase its uptake. Our chatbot advances The National Human Genome Research Institute’s call to leverage digital health-enabled genomics to increase public genomic literacy (Bonham and Green 2021) and address ethnocultural gaps in application of digital health technologies (Bombard et al. 2022).

However, as participants recommended, providing a voice-over feature would further increase access to the chatbot content for those who cannot read, which companies designing chatbots should address. The digital divide is closing whereby comparable rates of smartphone use were reported among Black (83%) and White (85%) Pew national survey respondents (Atske and Perrin 2021).

Gia was designed to foster the integration of genetic testing and counseling for LKD evaluation. Future research will examine use of the *APOL1* Gia chatbot in real-world settings using self-reported perceptions and log data. Our research team is implementing Gia into donor clinics to evaluate its impact on LKD candidates’ decision-making about *APOL1* genetic testing and donation (Smith et al. 2023). Critics’ fears that chatbots will dehumanize healthcare delivery and replace genetic counselors (Reddy et al. 2019) are unfounded because chatbots aid, rather than replace, human decision-making (Nazareth et al. 2021). Conversely, it is possible that providers may come to over-rely on chatbots. While some commentators fear that physicians’ growing reliance on artificial intelligence such as chatbots will crowd out physicians who do not use artificial intelligence (AI), this is unlikely as it will likely take concerted effort by healthcare organizations to invest in strategies including human-AI collaboration to repurpose roles for increased efficiencies (Sezgin 2023). Accordingly, our intent is to implement this digital health technology to increase healthcare efficiencies in genomic medicine by releasing time for transplant nephrologists to discuss *APOL1* genetic testing and living donation and foster patient-centered care delivery. Our future research will disseminate Gia to scale *APOL1* genetic testing and counseling for LKDs.

There are several strengths of this study. This was a multi-site study among LKDs, living donor kidney transplant recipients, and community members of African ancestry, which can foster transferability of findings. Our study provided novel insights into attitudes about chatbot use among individuals of African ancestry, about whom relatively little is known. The chatbot was rigorously developed in a patient-centered manner: Individuals from diverse racial/ethnic and educational backgrounds were involved in the process of designing Gia so that the chat would culturally resonate and be accepted by a broad population. The Scientific Advisory Board provided input that supported face and content validity, while the Community Advisory Board provided feedback and focus group participants of African ancestry supported face validity and cultural insights. We followed established methodologies for survey development, and conducted rigorous pre-testing of the chatbot content to ensure its clarity and comprehensiveness.

This study may have limitations. We cannot exclude the possibility that participants did not understand information. Because the research occurred primarily with individuals in urban areas, the views may not be transferrable to those in rural areas. Although more females participated in focus groups than males, the proportion of female LKDs mirrored that of LKDs nationally. However, the average age of our focus group participants was higher than the average age of LKDs. Some focus group participants may have been hesitant to fully express their views in a group setting. Although moderators were trained, some biases in moderating style across sites could have occurred.

## Conclusion

Our findings suggest that individuals of African ancestry perceived Gia as informative about *APOL1* genetic testing, neutral and unbiased, trustworthy, and culturally targeted to individuals of African ancestry. Future research will assess the impact of *APOL1* Gia on informed decision-making about *APOL1* genetic testing among LKD candidates of African ancestry. As a supplement to physician medical care, Gia may augment *APOL1* counseling and testing in LKD clinical evaluation.

### Supplementary information

Below is the link to the electronic supplementary material.

Supplementary file1 (DOCX 16 KB)

Supplementary file2 (DOCX 52.2 KB)

## Data Availability

The datasets generated during and/or analyzed during the current study are available from the corresponding author on reasonable request.

## Abbreviations

*APOL1*: *Apolipoprotein* L1
CAB: Community Advisory Board
Gia: Genetic Information Assistant
HIPAA: Health Insurance Portability and Accountability Act
LKD: Living kidney donor
NHW: Non-Hispanic White
OPTN: Organ Procurement and Transplant Network
PI: Principal investigator

## Declarations

### Declaration of AI and AI-assisted technologies in the writing process.

Absolutely no form of Generative Artificial Intelligence was used in the scientific writing of this manuscript.

## References

[CR1] Allouch M, Azaria A, Azoulay R (2021) Conversational agents: goals, technologies, vision and challenges. Sensors (Basel) 21(24). 10.3390/s21248448 10.3390/s21248448 PMC8704682 34960538

[CR2] Arriola KR, Powell CL, Thompson NJ, Perryman JP, Basu M (2014). Living donor transplant education for African American patients with end-stage renal disease. Prog Transplant.

[CR3] Atske S, Perrin A (2021) Home broadband adoption, computer ownership vary by race, ethnicity in the U.S. Pew Research Center. URL: https://www.pewresearch.org/short-reads/2021/07/16/home-broadband-adoption-computer-ownership-vary-by-race-ethnicity-inthe-u-s/. Accessed 1 May 2023

[CR4] Barrera M, Berkel C, Castro FG (2017). Directions for the advancement of culturally adapted preventive interventions: local adaptations, engagement, and sustainability. Prev Sci.

[CR5] Bickmore TW, Silliman RA, Nelson K (2013). A randomized controlled trial of an automated exercise coach for older adults. J Am Geriatr Soc.

[CR6] Bombard Y, Ginsburg GS, Sturm AC, Zhou AY, Lemke AA (2022). Digital health-enabled genomics: opportunities and challenges. Am J Hum Genet.

[CR7] Bonham VL, Green ED (2021). The genomics workforce must become more diverse: a strategic imperative. Am J Hum Genet.

[CR8] Caliskan Y, Lee B, Whelan A, Abualrub F, Lentine KL, Jittirat A (2022). Evaluation of genetic kidney diseases in living donor kidney transplantation: towards precision genomic medicine in donor risk assessment. Curr Transplant Rep.

[CR9] Creswell JW, Plano Clark VL (2007). Designing and conducting mixed methods research.

[CR10] Doshi MD, Gordon EJ, Freedman BI, Glover C, Locke JE, Thomas CP (2021). Integrating APOL1 kidney-risk variant testing in live kidney donor evaluation: an expert panel opinion. Transplantation.

[CR11] Dworkin MS, Lee S, Chakraborty A (2019). Acceptability, feasibility, and preliminary efficacy of a theory-based relational embodied conversational agent mobile phone intervention to promote hiv medication adherence in young HIV-positive African American MSM. AIDS Educ Prev.

[CR12] Fan X, Chao D, Zhang Z, Wang D, Li X, Tian F (2021). Utilization of self-diagnosis health chatbots in real-world settings: case study. J Med Internet Res.

[CR13] Freedman BI, Poggio ED (2021). APOL1 genotyping in kidney transplantation: to do or not to do, that is the question? (pro). Kidney Int.

[CR14] Friedman D, Pollack M (2011). Genetics of kidney failure and the evolving story of APOL1. J Clin Investig.

[CR15] Glanz K, Lewis F, Rimer B (1997) Health behavior and health education. Jossey-Bass

[CR16] Gordon EJ, Wicklund C, Lee J, Sharp RR, Friedewald J (2018). A national survey of transplant surgeons and nephrologists on implementing apolipoprotein L1 (*APOL1*) genetic testing into clinical practice. Prog Transplant.

[CR17] Gordon EJ, Amortegui D, Blancas I, Wicklund C, Friedewald J, Sharp RR (2018). African American living donors’ attitudes about APOL1 genetic testing: a mixed methods study. Am J Kidney Dis.

[CR18] Gordon EJ, Amortegui D, Blancas I, Wicklund C, Friedewald J, Sharp RR (2019). A focus group study on African American living donors’ treatment preferences, sociocultural factors, and health beliefs about apolipoprotein L1 genetic testing. Prog Transplant.

[CR19] Guest G, MacQueen K, Namey E (2012). Applied thematic analysis.

[CR20] Guidry JJ, Walker VD (1999). Assessing cultural sensitivity in printed cancer materials. Cancer Pract Nov-Dec.

[CR21] Ireland D, Bradford D, Szepe E (2021). Introducing Edna: a trainee chatbot designed to support communication about additional (secondary) genomic findings. Patient Educ Couns.

[CR22] Keith RE, Crosson JC, O'Malley AS, Cromp D, Taylor EF (2017). Using the consolidated framework for implementation research (CFIR) to produce actionable findings: a rapid-cycle evaluation approach to improving implementation. Implementation Sci : IS.

[CR23] Kramer HJ, Stilp AM, Laurie CC (2017). African Ancestry-specific alleles and kidney disease risk in Hispanics/Latinos. J Am Soc Nephrol.

[CR24] Kramer LL, Ter Stal S, Mulder BC, de Vet E, van Velsen L (2020). Developing embodied conversational agents for coaching people in a healthy lifestyle: scoping review. J Med Internet Res.

[CR25] Kreuter MW, Skinner CS (2000). Tailoring: what’s in a name?. Health Educ Res.

[CR26] Krueger  R (1994). Focus groups: a practical guide for applied research.

[CR27] Kumar V, Locke JE (2021). APOL1 genotyping in kidney transplantation: to do or not to do, that is the question? (contra). Kidney Int.

[CR28] Lazarin GA, Haque IS, Nazareth S (2013). An empirical estimate of carrier frequencies for 400+ causal Mendelian variants: results from an ethnically diverse clinical sample of 23,453 individuals. Genet Med.

[CR29] Lentine KL, Kasiske BL, Levey AS (2017). KDIGO Clinical practice guideline on the evaluation and care of living kidney donors. Transplantation.

[CR30] Luca S, Clausen M, Shaw A (2023). Finding the sweet spot: a qualitative study exploring patients’ acceptability of chatbots in genetic service delivery. Hum Genet.

[CR31] Muzaale AD, Massie AB, Wang MC (2014). Risk of end-stage renal disease following live kidney donation. JAMA.

[CR32] Nadkarni GN, Gignoux CR, Sorokin EP (2018). Worldwide frequencies of APOL1 renal risk variants. N Engl J Med.

[CR33] National Society of Genetic Counselors (2022) Professional status survey 2022: Executive summary. https://www.nsgc.org/Portals/0/Executive%20Summary%20Final%2005-03-22.pdf.2022. Accessed 30 Jan 2024

[CR34] Nazareth S, Nussbaum RL, Siglen E, Wicklund CA (2021). Chatbots & artificial intelligence to scale genetic information delivery. J Genet Couns.

[CR35] Owens OL, Felder T, Tavakoli AS (2019). Evaluation of a computer-based decision aid for promoting informed prostate cancer screening decisions among African American men: iDecide. Am J Health Promotion : AJHP.

[CR36] Parsa A, Kao WH, Xie D (2013). APOL1 risk variants, race, and progression of chronic kidney disease. N Engl J Med.

[CR37] Pereira J, Diaz O (2019). Using health chatbots for behavior change: a mapping study. J Med Syst.

[CR38] Ponathil A, Ozkan F, Welch B, Bertrand J, Chalil Madathil K (2020) Family health history collected by virtual conversational agents: an empirical study to investigate the efficacy of this approach. J Genet Couns. 10.1002/jgc4.1239 10.1002/jgc4.1239 32125052

[CR39] Rasouly HM, Balderes O, Marasa M (2023). The effect of genetic education on the referral of patients to genetic evaluation: findings from a national survey of nephrologists. Genet Med.

[CR40] Ray T (2018) Geisinger deploys ‘Gia’ chatbot to help genetic counselors manage MyCode participants. Genome Web. https://www.genomeweb.com/informatics/geisinger-deploys-gia-chatbot-help-genetic-counselors-manage-mycode-participants#.Xm1PfmhKg2w. Accessed 14 Mar 2020

[CR41] Reddy S, Fox J, Purohit MP (2019). Artificial intelligence-enabled healthcare delivery. J R Soc Med.

[CR42] Resnicow K, Baranowski T, Ahluwalia JS, Braithwaite RL (1999). Cultural sensitivity in public health: defined and demystified. Ethnicity Dis Winter.

[CR43] Riella L, Sheridan A (2015). Testing for high-risk APOL1 alleles in potential living kidney donors. Am J Kidney Dis.

[CR44] Schmidlen T, Sturm AC, Hovick S (2018). Operationalizing the reciprocal engagement model of genetic counseling practice: a framework for the scalable delivery of genomic counseling and testing. J Genet Couns.

[CR45] Schmidlen T, Schwartz M, DiLoreto K, Kirchner HL, Sturm AC (2019). Patient assessment of chatbots for the scalable delivery of genetic counseling. J Genet Couns.

[CR46] Sezgin E (2023). Artificial intelligence in healthcare: complementing, not replacing, doctors and healthcare providers. Digital Health Jan-Dec.

[CR47] Siglen E, Vetti HH, Lunde ABF (2022). Ask Rosa - the making of a digital genetic conversation tool, a chatbot, about hereditary breast and ovarian cancer. Patient Educ Couns.

[CR48] Simms M (2018) Say African American or Black, but first acknowledge the persistence of structural racism. Urban Wire. https://www.urban.org/urban-wire/say-african-american-or-black-first-acknowledge-persistence-structural-racism. Accessed 13 Jan 2024

[CR49] Smith JD, Agrawal A, Wicklund C (2023). Implementation of a culturally competent APOL1 genetic testing program into living donor evaluation: a two-site, non-randomized, pre-post trial design. BMJ Open.

[CR50] Tan JC, Gordon EJ, Dew MA (2015). Living donor kidney transplantation: facilitating education about live kidney donation-recommendations from a consensus conference. Clin J Am Soc Nephrol.

[CR51] Tong A, Sainsbury P, Craig J (2007). Consolidated criteria for reporting qualitative research (COREQ): a 32-item checklist for interviews and focus groups. Int J Qual Health Care.

