# Supplementary material for: Development of a culturally targeted chatbot to inform living kidney donor candidates of African ancestry about APOL1 genetic testing: a mixed methods study
Source: J Community Genet. 2024 Feb 13;15(2):205–16. doi: 10.1007/s12687-024-00698-8 (PMC11031529; doi:10.1007/s12687-024-00698-8)
Supplement: Supplementary file 1 — Supplementary file1 (DOCX 16 KB) [file 12687_2024_698_MOESM1_ESM.docx]

| **Supplemental Table 1. Self-Reported African Ancestry** | | | |
| --- | --- | --- | --- |
| **Characteristic** | **Total**  **N=54**  **n (%)** | **NU**  **n=30**  **n (%)** | **GU**  **n=24**  **n (%)** |
| **Awareness of biologically related family members with African ancestry** |  |  |  |
| Yes | 37 (68.5) | 22 (73.3) | 15 (62.5) |
| No | 17 (31.5) | 8 (26.7) | 9 (37.5) |
| **Who in family has African ancestry** |  |  |  |
| Not applicable | 17 (31.5) | 8 (26.7) | 9 (37.5) |
| Both parents | 12 (22.2) | 9 (30.0) | 3 (12.5) |
| Entire family | 9 (16.7) | 2 (6.7) | 7 (29.2) |
| Father | 6 (11.1) | 5 (16.7) | 1 (4.2) |
| Mother | 5 (9.3) | 3 (10.0) | 2 (8.3) |
| Self | 2 (3.7) | 1 (3.3) | 1 (4.2) |
| Black American | 1 (1.9) | 1 (3.3) | 0 (0) |
| Great (7x) grandfather | 1 (1.9) | 1 (3.3) | 0 (0) |
| Unsure | 1 (1.9) | 0 (0) | 1 (4.2) |
| **Ancestry** |  |  |  |
| African | 15 (27.8) | 11 (36.7) | 4 (16.7) |
| African American | 12 (22.2) | 5 (16.7) | 7 (29.2) |
| Black | 6 (11.1) | 4 (13.3) | 2 (8.3) |
| Black American | 3 (5.6) | 2 (6.7) | 1 (4.2) |
| South Africa | 2 (3.7) | 2 (6.7) | 0 (0) |
| Unknown | 2 (3.7) | 0 (0) | 2 (8.3) |
| African American, Native American, Jewish | 1 (1.9) | 0 (0) | 1 (4.2) |
| African and Irish | 1 (1.9) | 1 (3.3) | 0 (0) |
| African, German, Indian | 1 (1.9) | 1 (3.3) | 0 (0) |
| African and Indian | 1 (1.9) | 1 (3.3) | 0 (0) |
| BIPOC | 1 (1.9) | 0 (0) | 1 (4.2) |
| Cameroon and Nigeria | 1 (1.9) | 0 (0) | 1 (4.2) |
| Cameroon/Congo Bantu people | 1 (1.9) | 0 (0) | 1 (4.2) |
| Caribbean | 1 (1.9) | 0 (0) | 1 (4.2) |
| Jamaican | 1 (1.9) | 0 (0) | 1 (4.2) |
| Kenya | 1 (1.9) | 1 (3.3) | 0 (0) |
| Nigeria, Ghana, Cameroon | 1 (1.9) | 0 (0) | 1 (4.2) |
| Nigeria, France, Ireland, Scotland | 1 (1.9) | 0 (0) | 1 (4.2) |
| Sub Saharan descent | 1 (1.9) | 1 (3.3) | 0 (0) |
| No response | 1 (1.9) | 1 (3.3) | 0 (0) |
