# Supplementary material for: Development of a culturally targeted chatbot to inform living kidney donor candidates of African ancestry about APOL1 genetic testing: a mixed methods study
Source: J Community Genet. 2024 Feb 13;15(2):205–16. doi: 10.1007/s12687-024-00698-8 (PMC11031529; doi:10.1007/s12687-024-00698-8)
Supplement: Supplementary file 2 — Supplementary file2 (DOCX 52.2 KB) [file 12687_2024_698_MOESM2_ESM.docx]

**Supplemental Table 2. Gia chatbot suggested changes based on focus group feedback**

| **Suggested Change** | **Change Implemented?** | | **Rationale for NOT Implementing Change** | **Description of Change Made to Gia Chat** | **Solicited or Unsolicited?** |
| --- | --- | --- | --- | --- | --- |
| **Content** | | | | | |
| Increase the number of minutes stated at the beginning of the chat for how long it should take to review the chat | Yes | | Not applicable | The number of minutes for how long the chat should take was increased from “5-7 min” to “7-12 min” based on participants’ timed use at the beginning of the focus groups | Solicited |
| Add a resource with living donors speaking about their past experiences of donation and how they are doing now | Yes | | Not applicable | We added links to websites with living donor testimonials at the end of the chat | Unsolicited |
| Add more information for users to dive “deeper” into *APOL1* and living donation | Yes | | Not applicable | We added website links to resources and information about the *APOL1* gene and living donation to the end of the chat | Unsolicited |
| Make sure the language in the Gia chat is easy to understand, with straightforward terminology and not many medical terms | Yes | | Not applicable | We added a hover-over feature to provide definitions and explanations of medical terminology and lesser-known topics | Solicited |
| Add more detail about how the *APOL1* gene evolved over time | Yes | | Not applicable | We added information about the *APOL1* gene evolution in a hover-over feature in the chat | Solicited |
| Add statistics on how many people with *APOL1* gene variants develop chronic kidney disease | Yes | | Not Applicable | We added the following text to the chat: “The chance that someone in the general population with 2 *APOL1* risk variants will get kidney disease in their lifetime is up to 20% (or up to 20 people out of 100 people)” | Unsolicited |
| Add the text, “The *APOL1* test does not disqualify you from being a donor. This test provides additional information for you and the transplant team to make a recommendation.” | No | | This text was not added to the chat because it is up to the transplant clinical team, and not the Gia chat, to determine whether the potential living donor will be eligible to donate | Not applicable | Unsolicited |
| Add a question at the end of the chat: "Will I be able to donate if I have 2 *APOL1* gene variants?" | No | | This topic is best left for direct discussion between the donor candidate and the nephrologist, per guidance from nephrologist and genetic counselor research collaborators | Not applicable | Solicited |
| Remove the question and answer about the likelihood of children having *APOL1* variants based on how many risk variants their parents have because the information is not relevant to the chat and living donation | No | | We retained this question and information as an option for users to select because participants varied in their desire for this information and some felt the information could be important to younger living donors who may want to know the answer if they plan to have children | Not Applicable | Unsolicited |
| Add information to the chat about the background and development of the *APOL1* genetic test | No | | This information was outside the scope of the Gia chat | Not applicable | Unsolicited |
| **Function** | | | | |  |
| Add a feature that enables the user to return to the chat where they left off, instead of sending them back to the beginning | Yes | | Not applicable | We added this functionality to support usability | Unsolicited |
| Add more detail to instruct the user on how to find the chat menu and menu options | Yes | | Not applicable | We added language in the chat to remind participants about the chat menu, including what the menu icon looks like, how to find it, and that options are available in the menu (e.g., change font size, change chat speed) | Solicited |
| Allow users to access the Gia chat content so that it can be reviewed again later and shared with their healthcare providers | Yes | | Not applicable | We added an option for users to enter their email to have the Gia chat content that they reviewed emailed to them | Unsolicited |
| Add a voiceover/read-aloud feature to the Gia chatbot | No | | Invitae did not offer voiceover functionality with this Gia chatbot | Not applicable | Unsolicited |
| Make the chatbot more interactive by animating the Gia avatar and having it talk to the user | No | | Invitae did not have the capability to host animation or audio in this Gia chatbot | Not applicable | Unsolicited |
| Add the ability for the user to type in their own questions and have Gia answer the questions in the chat | No | | Implementation of a free-text-based question feature is most effective when there are a very large number and variety of commonly-anticipated questions. This feature would have required extensive development and could have resulted in many participants getting no response (if their question was not matched to an anticipated question and response pair) or an incorrect or imprecise response | The research team determined that a more precise and efficient option was to provide participants with a list of questions to choose from and give exact responses to those questions in the chat. This solution was ideal because the number of commonly asked questions was small (based on our prior research) | Unsolicited |
| Add an option for users to type their own notes and questions within the chat, to share with their nephrologist later | No | | Invitae did not offer users the functionality to freely type notes into the Gia chat. However, we added a comment in the chat to let users know to have a pencil and paper handy to write down notes and questions that arise during the chat so they can discuss them with their nephrologist later | Not applicable | Unsolicited |
| Enable users to contact a member of the research team, e.g., through email, if they have questions about the chat | No | | The research team did not have the capacity to support this feature | Not applicable | Unsolicited |
| **Appearance and Format** | | | | |  |
| Make the default text size larger in the Gia chat | Yes | | Not applicable | The default text size of the Gia chat was made larger | Unsolicited |
| Make the hover-over text more noticeable | Yes | | Not applicable | We made the hover-over text bold, underlined, and in a different color to stand out | Solicited |
| Make the overall text of the Gia chat and the start button darker for easier reading | No | | Invitae’s color scheme for the Gia chat could not be modified | Not applicable | Unsolicited |
| Change the Gia avatar to a non-human image such as a cartoon image of a kidney | No | | The Gia chatbot is meant to mimic a chat experience that the user would have with another person, therefore, we maintained a ‘human’ image for the Gia avatar | Not applicable | Solicited |
| Make the “Menu” more visible by adding an animation of a waving hand | No | Invitae does not offer moving animations | | Not applicable | Solicited |
| **Response Options** | | | | |  |
| Change the Gia response “Great. Let's move on” to a different phrase because it comes across as abrupt and curt | Yes | | Not Applicable | We changed the phrase to “Okay that makes sense” to be more user-centered | Solicited |
| Change the response option “sounds good” located after the discussion about risks and benefits of testing (later changed to ‘pros and cons’ of testing) | Yes | | Not Applicable | We replaced “sounds good” with ‘‘That’s really good to know” because the original response did not reflect how people would typically react to hearing about risks | Unsolicited |
| Change all Gia response options to ‘Next’ or ‘Skip’ for users to move through the chat more efficiently | No | We retained the chat response options to uphold the conversational intent and feel of the chat | | Not applicable | Solicited |
| **Pathway/Branching** | | | | |  |
| Remove the branching pathway that begins with “that makes sense” and “thumbs up” because the same chat content follows, regardless of the response chosen | Yes | Not applicable | | Branching for this portion was removed, and the singular response “that makes sense” was maintained | Solicited |
| Remove all branching pathways in the Gia chat so that all users receive the same information | No | We maintained the branching pathways to personalize the chat experience according to each user’s learning needs. Topics addressed in the branching pathways include additional information about kidney disease, how genes work, and how long the medical community has known about *APOL1*, among other topics | | Not applicable | Unsolicited |
| Create additional response options that users can select to bypass content in the chat that they do not want to review | No | We retained the original structure of the chat to ensure that all users receive foundational information about *APOL1* and kidney disease | | Not applicable | Unsolicited |
| **Cultural Competency** | | | | |  |
| Aim to be informative rather than persuasive (neutral) | Yes | Not Applicable | | No changes were made because respondents confirmed that Gia was neutral and not persuasive as it is | Solicited |
| Include statistics about the number of African American/Black living kidney donors | Yes | Not Applicable | | We added statistics about the number of African American/Black living kidney donors to help with normalizing donation in the African American/Black community | Unsolicited |
| Add statistics about the number of people on the waiting list who need a kidney transplant versus the number of people who receive them | Yes | Not Applicable | | We added statistics to the question section at the end of the chat | Solicited |
| Provide a “simple” statement about the purpose of Gia at the beginning of the chat, instead of immediately presenting  information about *APOL1*, to inform and “reassure” participants about Gia’s intent | Yes | Not Applicable | | We added the purpose of Gia within the first few lines of the chat, before covering information about *APOL1;* it states, “I was designed to talk with you about genetic information, and your healthcare team has helped make sure that the info I share is accurate.” | Unsolicited |
| Explain how having information from *APOL1* testing can benefit living donors with African ancestry | Yes | Not Applicable | | Gia addresses how the *APOL1* genetic test can help living donor candidates become better prepared about donation by better understanding their risks of kidney disease post-donation | Solicited |
| Discuss all racial/ethnic groups affected by *APOL1*, and do not place all emphasis on people of African ancestry | Yes | Not Applicable | | Gia’s focus is on living donor candidates of African ancestry. However, Gia includes risk statistics for other racial/ethnic groups | Unsolicited |
| State “Caribbean” instead of “Afro-Caribbean” as one of the groups of the African diaspora | Yes | Not Applicable | | We changed “Afro-Caribbean” to “Caribbean” in the following:  “APOL1 risk variants are most commonly found in people with African ancestry, like Blacks/African Americans, Caribbeans, and Central/South Americans” | Solicited |
| Make the Gia dialogue more “natural” and “familiar with an African American audience” by changing the response option “Okay”, which can feel “very short and snippy” | Yes | Not Applicable | | We replaced the response options stating “Okay” to alternate responses, such as:   - “That makes sense” - “Yes, I understand” - “Okay, that makes sense” - “That's good to know” - “Thanks, I got it” | Solicited |
| Destigmatize *APOL1* risk variants by conveying a positive message about why people of African ancestry have a higher chance of having the risk variants. | Yes |  | | Information was added to Gia to explain that *APOL1* risk variants evolved thousands of years ago to protect people in Sub-Saharan Africa against sleeping sickness, and an explanation about sleeping sickness was added in a hover-over. | Solicited |
| Allow Gia to look like whatever race you self-identify in the chatbot | No | Invitae did not have the capability to add the option for users to change the Gia avatar in this Gia chatbot | | Not Applicable | Solicited |
| Along with testimonials of African American living donors, provide transplant-center specific statistics and national statistics about living donation and transplantation | Yes and No | Gia is intended for use across all transplant programs. Including transplant-center specific statistics would make scaling up the use of Gia less feasible in future studies | | We added URL links to websites that provide national statistics | Unsolicited |
| Discuss success stories of people of African ancestry who donated with two *APOL1* risk variants | Yes and No | We did not include testimonials of donors of African ancestry with two *APOL1* risk variants because none were available | | Not Applicable  We added testimonials of donors of African ancestry | Solicited |
